# Population pharmacokinetic and exposure‐response analyses from ALTA‐1L: Model‐based analyses supporting the brigatinib dose in *ALK*‐positive NSCLC

**DOI:** 10.1111/cts.13231

**Published:** 2022-02-08

**Authors:** Neeraj Gupta, Karen L. Reckamp, David R. Camidge, Huub J. Kleijn, Aziz Ouerdani, Francesco Bellanti, John Maringwa, Michael J. Hanley, Shining Wang, Pingkuan Zhang, Karthik Venkatakrishnan

**Affiliations:** ^1^ Takeda Development Center Americas, Inc. Lexington Massachusetts USA; ^2^ Samuel Oschin Cancer Center Cedars‐Sinai Medical Center Los Angeles California USA; ^3^ University of Colorado Cancer Center Aurora Colorado USA; ^4^ Certara Princeton New Jersey USA; ^5^ Millennium Pharmaceuticals, Inc., a wholly owned subsidiary of Takeda Pharmaceutical Company Limited Cambridge Massachusetts USA

## Abstract

The ALK in Lung Cancer Trial of brigAtinib in First Line (ALTA‐1L) compared brigatinib versus crizotinib in anaplastic lymphoma kinase (ALK) inhibitor‐naive patients with *ALK*+ non‐small cell lung cancer (NSCLC). A population pharmacokinetic (PK) model was used to estimate brigatinib exposures for exposure‐efficacy and exposure‐safety analyses in ALTA‐1L. A previously developed population PK model for brigatinib was applied to estimate brigatinib PK parameters. Relationships between static (time‐independent) and dynamic (time‐varying) exposure metrics and efficacy (progression‐free survival [PFS], objective response rate [ORR], and intracranial ORR [iORR]) and safety outcomes (selected grade ≥2 and grade ≥3 adverse events [AEs]) were evaluated using logistic regression and time‐to‐event analyses. There were no meaningful differences in brigatinib PK in the first‐line and second‐line settings, supporting use of the previous population PK model for the first‐line population. Exposure‐response analyses showed no significant effect of time‐varying brigatinib exposure on PFS. Brigatinib exposure was not significantly related to ORR, but higher exposure was associated with higher iORR (odds ratio: 1.13, 95% confidence interval: 1.01–1.28, *p* = 0.049). Across the observed median exposure (5th–95th percentile) at steady state for 180 mg once daily, the predicted probability of iORR was 0.83 (0.58–0.99). AEs significantly associated with higher exposure were elevated lipase (grade ≥3) and amylase (grade ≥2). Time to first brigatinib dose reduction was not related to exposure. These results support the benefit‐risk profile of first‐line brigatinib 180 mg once daily (7‐day lead‐in dose at 90 mg once daily) in patients with *ALK*+ NSCLC.

**Study Highlights**

**WHAT IS THE CURRENT KNOWLEDGE ON THE TOPIC?**

A population pharmacokinetic (PK) analysis conducted using data from five brigatinib clinical studies, including healthy volunteers and patients with cancer, demonstrated that age, sex, race, body weight, mild or moderate renal impairment, and mild hepatic impairment had no clinically meaningful effects on brigatinib PKs. Exposure‐response analyses conducted for efficacy and safety outcomes using brigatinib phase I and II trial data predicted clinically meaningful dose‐related improvements in progression‐free survival (PFS), intracranial PFS, and overall survival over the brigatinib dose range from 90 to 180 mg.

**WHAT QUESTION DID THIS STUDY ADDRESS?**

Do exposure‐response results quantitatively support a benefit/risk profile of the 180 mg once daily (with 7‐day lead‐in at 90 mg once daily) brigatinib dosing regimen for first‐line treatment of patients with anaplastic lymphoma kinase‐positive (*ALK*+) non‐small cell lung cancer (NSCLC)?

**WHAT DOES THIS STUDY ADD TO OUR KNOWLEDGE?**

The brigatinib systemic exposures in patients receiving first‐line brigatinib were similar to those observed for second‐line brigatinib. Brigatinib exposure was not a discernible predictor of PFS or objective response rate (ORR). A statistically significant relationship was identified between brigatinib exposure and intracranial ORR (iORR), with higher exposure associated with higher iORR. With the exceptions of grade ≥2 amylase and grade ≥3 lipase elevations, no significant relationship was identified between brigatinib exposure and adverse event incidence or time to first dose reduction.

**HOW MIGHT THIS CHANGE CLINICAL PHARMACOLOGY OR TRANSLATIONAL SCIENCE?**

These descriptive exposure‐efficacy and exposure‐safety results supported regulatory review of the brigatinib dosing regimen for first‐line treatment of patients with *ALK*+ NSCLC.

## INTRODUCTION

Brigatinib is an oral tyrosine kinase inhibitor (TKI) with potent activity against a broad range of anaplastic lymphoma kinase (*ALK*) gene resistance mutations, including the fusion protein EML4‐ALK found in ~ 5% of patients with non‐small cell lung cancer (NSCLC).^1^, ^2^, ^3^ Results of a randomized, multicenter phase II trial (ALK in Lung Cancer Trial of AP26113 [ALTA], NCT02094573) supported the initial approval of brigatinib in advanced *ALK*‐positive (*ALK*+) NSCLC that progressed on crizotinib.^4^, ^5^ The recommended brigatinib dosing regimen is 180 mg once daily (q.d.), with a 7‐day lead‐in at 90 mg q.d. This dosing regimen mitigates the risk of early onset pulmonary events (interstitial lung disease [ILD] and pneumonitis) observed in phase I and II trials at starting doses greater than 90 mg q.d.^3^, ^4^ In ALTA, this dosing regimen provided an independent review committee (IRC)‐assessed confirmed objective response rate (ORR) of 56%; median IRC‐assessed progression‐free survival (PFS) was 16.7 months.^5^ IRC‐assessed intracranial ORR (iORR) in patients with measurable baseline brain metastases was 67%; median duration of response was 16.6 months.^5^, ^6^ The most common brigatinib‐related adverse events (AEs) in ALTA were gastrointestinal AEs and increased blood creatine phosphokinase (CPK).^5^

Brigatinib was subsequently approved for first‐line treatment of *ALK*+ NSCLC based on results of the phase III ALK in Lung Cancer Trial of brigAtinib in First Line (ALTA‐1L; NCT02737501) comparing the efficacy and safety of brigatinib versus crizotinib in patients with advanced ALK inhibitor‐naive *ALK*+ NSCLC.^7^, ^8^ In ALTA‐1L, brigatinib met the prespecified threshold for superiority over crizotinib at the first interim analysis (hazard ratio [HR]: 0.49, 95% CI: 0.33–0.74, *p* < 0.001), which was maintained at the second interim analysis with median follow‐up of 24.9 months.^8^ The safety profile of brigatinib was consistent with that reported in ALTA; the most common AEs were gastrointestinal AEs, increased CPK, cough, hypertension, and increased aspartate aminotransferase (AST).^8^ Early onset pulmonary events were reported in 3% of brigatinib‐treated patients.^8^

Brigatinib is rapidly absorbed following oral administration, without clinically relevant food effects, and systemic exposures increase in a dose‐proportional manner over the 60‐ to 240‐mg dose range.^2^, ^9^, ^10^ After administration of 180 mg q.d., the mean plasma elimination half‐life is 25 h.^9^

A population pharmacokinetic (PK) analysis was conducted using data from 105 healthy volunteers and 337 patients with cancer enrolled across five clinical studies, including ALTA.^11^ A three‐compartment model with transit absorption compartments best described brigatinib plasma concentrations following single and multiple doses. Covariate analyses demonstrated that age, sex, race, body weight, mild or moderate renal impairment, and mild hepatic impairment had no clinically meaningful effects on brigatinib PKs. However, dose reduction is recommended for patients with severe renal^12^ and severe hepatic impairment (Child‐Pugh class C)^9^ based on results from dedicated renal and hepatic impairment studies. Additionally, exposure‐response analyses were previously conducted for efficacy and safety outcomes using phase I and II trial data and supported the recommended clinical dosage.^3^, ^4^, ^13^

During the review of any drug, the US Food and Drug Administration recommends conducting supportive exposure‐response analyses to assess whether the approved dosing regimen and dose reduction recommendations need optimization.^14^ Hence, exposure‐response analysis was conducted to support brigatinib dosage in the first‐line setting. In this study, the previously developed population PK model^11^ was applied to concentration data from patients receiving first‐line brigatinib in ALTA‐1L to estimate individual brigatinib PK parameters, and static (time‐independent) and dynamic (time‐varying) brigatinib exposure metrics were subsequently derived to evaluate exposure‐response relationships of clinical efficacy outcomes, selected AEs, and time to first brigatinib dose reduction.

## METHODS

### Data collection

The population PK and exposure‐response analyses evaluated data from the brigatinib arm (180 mg q.d. with a 7‐day lead‐in at 90 mg q.d.) of the ALTA‐1L trial (second interim analysis, data cutoff date: June 28, 2019).^8^ Data from the crizotinib arm (250 mg twice daily) of the study were included only for comparison purposes in the exposure‐response analyses. Blood samples for measurement of brigatinib plasma concentrations were obtained predose on day 1 of cycle one (28 day/cycle); predose and 1, 4, and 6 to 8 h postdose on day 1 of cycle two; and predose and any time between 1 and 8 h postdose on day 1 of cycles three, four, and five. Plasma samples were analyzed for brigatinib concentrations using a validated liquid chromatography/tandem mass spectrometry assay with a lower limit of quantification of 5 ng/mL. Efficacy assessments were based on computed tomography or magnetic resonance imaging performed at screening, every 8 weeks through cycle 14, and then every 12 weeks through treatment discontinuation.^8^ Safety data were collected as they were reported.

### Population pharmacokinetic modeling and model evaluation

The previously developed population PK model^11^ was used for population PK analyses of ALTA‐1L data. The previous analysis provided a robust and comprehensive characterization of brigatinib PK and sources of variability. Importantly, the previous analysis included 202 patients with *ALK*+ NSCLC that progressed on other ALK TKIs, representing a similar patient population to patients receiving first‐line brigatinib in ALTA‐1L. These considerations supported a Bayesian re‐estimation approach for deriving individual brigatinib PK parameters in ALTA‐1L using the previously reported model, and the model was applied without modification. In brief, the population PK model described brigatinib PK using a three‐compartment model with linear distribution and elimination kinetics and first‐order absorption preceded by a set of transit compartments.^11^ The model included albumin concentration as a covariate on apparent oral clearance (CL/F). Bayesian re‐estimation was performed by setting initial model conditions to values of fixed effects and random effect variances previously estimated,^11^ allowing for estimation of individual PK parameters based on the PK observations in the ALTA‐1L dataset.

Diagnostic plots were created to compare brigatinib PK in patients in ALTA‐1L (first‐line setting) and ALTA (second‐line setting). Prediction‐corrected visual predictive checks showed the model‐predicted time course of median and 2.5 and 97.5 percentiles of predicted brigatinib concentrations based on 1000 simulated replicates of the analysis dataset overlaid with individual ALTA‐1L data. In exploratory analyses, relationships between covariates of interest and model‐based exposure estimates were evaluated using linear regression models with each covariate as a predictor.

### Exposure‐efficacy analyses

The exposure‐efficacy analyses related reported efficacy outcomes (blinded IRC [BIRC]‐assessed PFS, ORR, and iORR) to individual brigatinib exposures predicted using individual estimated PK parameters from the population PK model. Model‐based estimates of CL/F and actual dosing history were used to derive exposure metrics of interest. Several exposure metrics were considered, including both static and dynamic area under the concentration‐time curve (AUC) estimates (Methods S1).

First, the relationship between PFS and two static exposure metrics, time‐averaged AUC between the last two disease assessment scans preceding progression or censoring and time‐averaged AUC until progression, was evaluated. These exposure‐PFS relationships were characterized by a proportional hazard (PH) model relating exposure to the hazard of PFS. The hazard function was expressed as:

(1) $$\lambda \left(t\right)=\lambda_{0}\left(t\right)e^{\beta^{T}X_{i}}$$

where *λ*_0_ (*t*) is the baseline hazard function and *X_i_* is a vector of predictor variables for an individual patient. The parameter vector *β* was estimated by maximum partial likelihood and captures the effect of exposure and/or additional covariates on the hazard of disease progression.

Next, the relationship between PFS and two dynamic exposure metrics, daily time‐varying AUC between the last two disease assessment scans preceding progression or censoring and daily time‐varying AUC until progression, was evaluated. These analyses used a time‐dependent covariate Cox PH model relating time‐dependent exposure to PFS. The hazard function was expressed as:

(2) $$\lambda \left(t\right)=\lambda_{0}\left(t\right)e^{\beta_{1}\times AUCD_{t}+\beta^{T}X_{i}}$$

where model parameters were as above in Equation 1, except that exposure (*AUCD_t_*) was a time‐dependent predictor rather than a constant for each patient.

For each static exposure‐PFS analysis, Kaplan‐Meier (KM) plots of PFS probability over time were generated and stratified by brigatinib exposure quartiles. Crizotinib PFS curves were overlaid for comparison.

For ORR and iORR, each patient was classified as a responder (i.e., achieving confirmed complete or partial systemic ORR or iORR response) or a nonresponder. Relationships between ORR and iORR and brigatinib exposure were analyzed using two static exposure metrics: time‐averaged AUC between the last two disease assessment scans preceding best confirmed response and time‐averaged AUC until best confirmed response (ORR or iORR). Exposure–clinical response relationships were characterized by logistic regression models. Predicted logistic regression curve and observed response rate in each brigatinib exposure quartile or tertile (along with associated 95% CIs) were plotted.

### Exposure‐safety analyses

Exposure‐safety relationships were characterized using the static exposure metric of time‐averaged AUC to the first occurrence of an event (or to the end of treatment if no event). AEs of interest selected for analysis included eight grade two or higher AEs (increased alanine aminotransferase [ALT], AST, or amylase; hyperglycemia; hypertension; bradycardia; rash; and pulmonary events [pneumonitis or ILD]), five grade three or higher AEs (increased CPK, AST, ALT, amylase, and lipase), and a composite grade three or higher AE end point that included grade three or higher CPK, AST, ALT, amylase, or lipase elevations; hyperglycemia; hypertension; bradycardia; rash; and pulmonary events. An additional exposure metric of time‐averaged AUC across days 8 to 14 of cycle one was also evaluated to represent exposure early in the period after the brigatinib dose increase from 90 to 180 mg q.d. in order to explore potential associations with AEs.

AEs were included in the analysis if they occurred any time between the first day of brigatinib dosing and 30 days after the last dose. AEs were defined according to the National Cancer Institute Common Toxicity Criteria for Adverse Events version 4.03. The relationship between time‐averaged exposure and AE probability was examined using the same logistic regression models described for the exposure–clinical response analyses. Probability of an AE was plotted against exposure, with observed probabilities calculated by exposure quartiles.

The relationship between brigatinib exposure and time to first brigatinib dose reduction was also explored. A KM plot of time to first brigatinib dose reduction was generated, with survival curves binned by brigatinib exposure quartiles, where exposure was defined as the time‐averaged AUC to the first occurrence of dose reduction.

### Covariate analysis

If exposure was identified as a statistically significant (*p *= 0.05) predictor of the efficacy or safety end point of interest, a covariate model was developed. Covariate evaluation was conducted in a stepwise manner, applying an iterative forward addition (*p* < 0.05) and backward elimination (*p* > 0.01) model selection strategy. Exposure‐efficacy model covariates included sex, race, Eastern Cooperative Oncology Group (ECOG) performance status (0, 1, or 2), prior chemotherapy, presence of brain metastases, and smoking status (categorical covariates) and age, and log sum of baseline target lesions (continuous covariates). Covariate analysis was not conducted for the exposure‐efficacy model of iORR. Covariates for exposure‐safety models included age (continuous), sex, race, ECOG performance status, and prior chemotherapy (categorical).

### Analysis software

The population PK analysis was performed using nonlinear mixed effects modeling (NONMEM version 7.3; ICON Development Solutions, Hanover, MD, USA)^15^ running under PsN (Perl‐speaks‐NONMEM, version 4.8.1 or later). Data management, exposure‐response analyses, and evaluation of results were performed using R (version 3.5.2 or higher).^16^

## RESULTS

### Datasets

In ALTA‐1L, the intent‐to‐treat population for the brigatinib arm included 137 patients with *ALK*+ NSCLC.^7^, ^8^ Of these, 13 patients did not have quantifiable brigatinib concentrations and one patient did not receive brigatinib. Thus, 123 patients were included in the population PK analysis (providing a total of 1069 brigatinib PK samples) and in the exposure‐response analyses of efficacy (PFS, ORR [by BIRC]) and safety. Forty‐seven brigatinib‐treated patients had intracranial central nervous system metastases by BIRC assessment at baseline, five of whom had no brigatinib concentration data available. Therefore, 42 patients were included in the iORR exposure‐efficacy dataset. Demographic and baseline characteristics of the 123 PK‐evaluable patients in ALTA‐1L were similar to those of the 201 patients from ALTA included in the initial population PK analysis^11^ (Table 1).

**TABLE 1:** Baseline Characteristics for patients with *ALK*+ NSCLC in ALTA‐1L and ALTA

Continuous covariates, median (range)	ALTA−1L *n* = 123	ALTA *n* = 201
Age, years	57 (27–85)	53 (18–82)
Albumin, g/L^a^	41 (24–48)	36 (20–47)
ALT, U/L	20 (5–118)	30 (5–129)
AST, U/L	20 (9–111)	26 (10–88)
Bilirubin, μmol/L	8 (3–34)	9 (1–22)
Body weight, kg	67 (43–111)	70 (41–172)
eGFR, mL/min/1.73 m^2^	92 (40–178)	83 (37–278)
Log sum target lesions at baseline, mm^b^	3.92 (2.34–5.4)^c^	_
Categorical covariates, *n* (%)		
Sex		
Male	63 (51.2)	85 (42.3)
Female	60 (48.8)	116 (57.7)
Race		
White	68 (55.3)	133 (66.2)
Asian	53 (43.1)	64 (31.8)
Other	2 (1.6)	4 (2.0)
ECOG status,^b^ 0/1/2	49 (39.8)/69 (56.1)/5 (4.1)	_
Prior chemotherapy,^b^ No/Yes	88 (71.5)/35 (28.5)	_
Brain metastases at baseline,^b^ No/Yes	87 (70.7)/36 (29.3)	_

Abbreviations: *ALK*+, anaplastic lymphoma kinase positive; ALT, alanine aminotransferase; ALTA‐1L, Anaplastic Lymphoma Kinase in Lung Cancer Trial of brigAtinib in First Line; AST, aspartate aminotransferase; ECOG, Eastern Cooperative Oncology Group; eGFR, estimated glomerular filtration rate; NSCLC, non‐small cell lung cancer; PK, pharmacokinetic; U/L, units per liter.

^a^ Three patients in the ALTA‐1L trial were missing data on albumin.

^b^ Sum of target lesions at baseline, ECOG status, prior chemotherapy status, presence of brain metastases, and smoking status were included as covariates only in the exposure‐response analyses and are therefore not reported for the ALTA population (which was included only for assessment of the population PK model).

^c^ One patient in ALTA‐1L had missing data for log sum of target lesions at baseline.

### Population pharmacokinetic model assessment

Model‐predicted individual brigatinib concentrations were consistent with observed concentrations in ALTA‐1L (Figure S1), indicating that the population PK model could adequately describe observed PK data for first‐line brigatinib in individual patients with *ALK+* NSCLC. The conditional weighted residuals (CWRES) were normally distributed and showed no trend when related to population‐predicted concentrations or time (Figure S2), further supporting the ability of the model to describe ALTA‐1L data. Model‐predicted population brigatinib concentrations were slightly underpredicted compared with observed concentrations. A prediction‐corrected visual predictive check suggested slight underprediction of the data, but general agreement between predicted and observed concentrations (Figure S1E).

**FIGURE 1:**
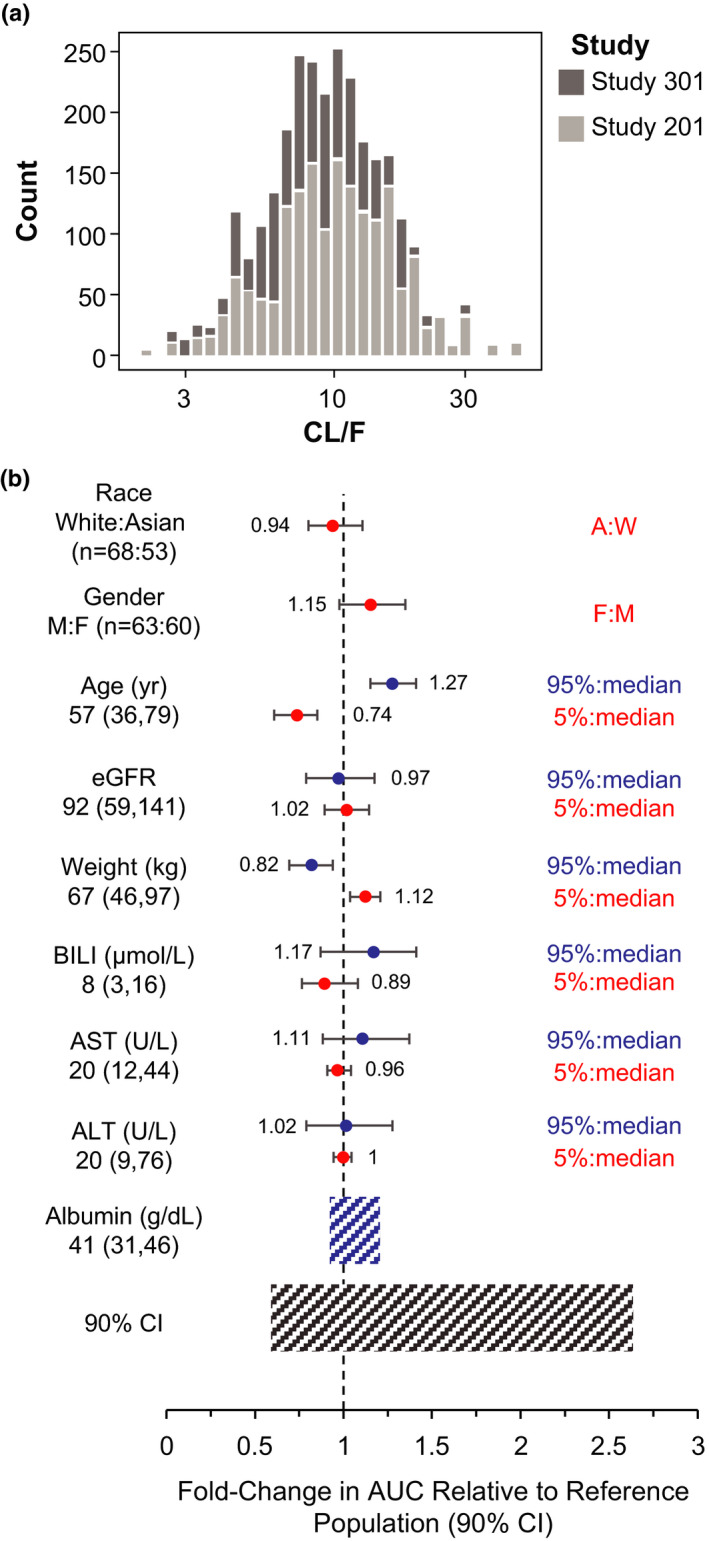
(a) Histogram comparing individual CL/F estimates between ALTA‐1L and ALTA. To further justify the ability of the model to accurately predict exposure in patients in ALTA‐1L, distributions of post hoc CL/F estimates were compared for patients in ALTA and ALTA‐1L. CL/F estimates were similar between the trials, confirming that the Bayesian re‐estimation approach was an appropriate method to obtain individual CL/F and post hoc exposure estimates for patients in ALTA‐1L. (b) Brigatinib exposure (AUC) following 180 mg once daily stratified by covariates of interest. The relationship between covariates (continuous: age, body weight, ALT, AST, bilirubin, and eGFR; categorical: sex and race) and model‐based exposure estimates was explored using linear regression models with each covariate as a predictor. Comparison of post hoc brigatinib AUC confirmed the lack of clinically meaningful effects of the covariates on brigatinib systemic exposure compared with overall exposure variability. *Black vertical line and values* at the base of the figure refer to the predicted AUC of brigatinib in a typical patient with baseline albumin of 41 g/dL. *Black shaded bar* illustrates the 5th to 95th percentile exposure range across the entire population expressed as a ratio relative to the reference exposure in a typical patient. *Blue shaded bar* represents the influence of baseline albumin on exposure. *Lines* above the blue shaded bar represent the different groups for the covariates listed at the left. For continuous covariates, the two *dots* represent the ratio of exposure of the 95th percentile covariate value versus the median and 5th percentile versus the median. For categorical covariates, *dots* represent the ratio of exposure of the categories versus the reference (most common) category. The *horizontal bars* on the two sides of the dots represent the corresponding 90% CI. A, Asian; ALT, alanine aminotransferase; AST, aspartate aminotransferase; AUC, area under the concentration‐time curve; BILI, bilirubin; CI, confidence interval; CL/F, apparent oral clearance from the central compartment; eGFR, estimated glomerular filtration rate; F, female; M, male; W, White

Post hoc estimates of brigatinib PK parameters were not meaningfully different for the ALTA‐1L and ALTA populations (Table 2). The distributions (Figure 1a; Figure S3) of individual PK parameter estimates did not meaningfully differ between the two trials, further verifying the Bayesian re‐estimation approach for obtaining individual PK parameters and exposure estimates using the previous population PK model. Daily steady‐state exposure based on the brigatinib maintenance dose of 180 mg q.d. (AUC) was estimated for each ALTA‐1L patient. The geometric mean post hoc AUC (5th and 95th percentiles) was 21.3 (10.1, 44.6) μg·h/mL.

**TABLE 2:** PK parameter estimates for patients from ALTA‐1L and ALTA based on the population PK model

Parameter	Geometric mean (5th and 95th percentiles of distribution)	
	ALTA−1L (*n* = 123)	ALTA (*n* = 201)
CL/F, L/h	8.45 (4.04, 17.8)	9.90 (4.06, 23.0)
V1/F, L	160 (69.0, 302)	199 (86.0, 468)
Q2/F, L/h	12.6 (12.6, 12.6)	12.6 (12.6, 12.6)
V2/F, L	118 (46.5, 283)	124 (78.3, 259)
Q3/F, L/h	2.67 (2.67, 2.67)	2.67 (2.67, 2.67)
V3/F, L	78.5 (78.5, 78.5)	78.5 (78.5, 78.5)
Transit compartments, *n*	2.76 (1.71, 5.37)	2.72 (1.85, 4.46)
Mean transit time, h	1.01 (0.538, 2.36)	0.998 (0.584, 1.92)
Albumin covariate effect on CL/F	1.03 (0.874, 1.13)	0.939 (0.758, 1.09)

Abbreviations: ALTA‐1L, Anaplastic Lymphoma Kinase in Lung Cancer Trial of brigAtinib in First Line; CL/F, apparent oral clearance from the central compartment; PK, pharmacokinetic; Q2/F, intercompartmental clearance between the central and first peripheral compartments; Q3/F, intercompartmental clearance between the central and second peripheral compartments; V1/F, apparent central volume of distribution; V2/F, apparent volume of distribution of the first peripheral compartment; V3/F, apparent volume of distribution of the second peripheral compartment.

The relationship between covariates of interest and post hoc AUC estimates was explored using linear regression models with each covariate as a predictor. None of the covariates examined had clinically meaningful effects on brigatinib exposure in ALTA‐1L. For continuous covariates (age, estimated glomerular filtration rate, body weight, total bilirubin, AST, and ALT), the magnitudes of relative difference in predicted AUC at the 5th and 95th percentiles relative to the predicted AUC at the median of individual covariate values were all below 30% (Figure S4), well below the variability in post hoc AUC in the overall ALTA‐1L population (5th and 95th percentiles were −60% to +263% relative to predicted AUC for a typical patient with an albumin level of 41 g/L; Figure S4). Similarly, for categorical covariates (sex and race), the magnitude of relative difference in predicted AUC for each stratum within the covariate compared with the most common stratum was less than 20% (Figure S5). For all covariates, the magnitude of effect on the brigatinib exposures was within the overall range of exposures, confirming the lack of clinically meaningful effects of these covariates on brigatinib systemic exposure (Figure 1b).

### Exposure‐efficacy analysis

**FIGURE 2:**
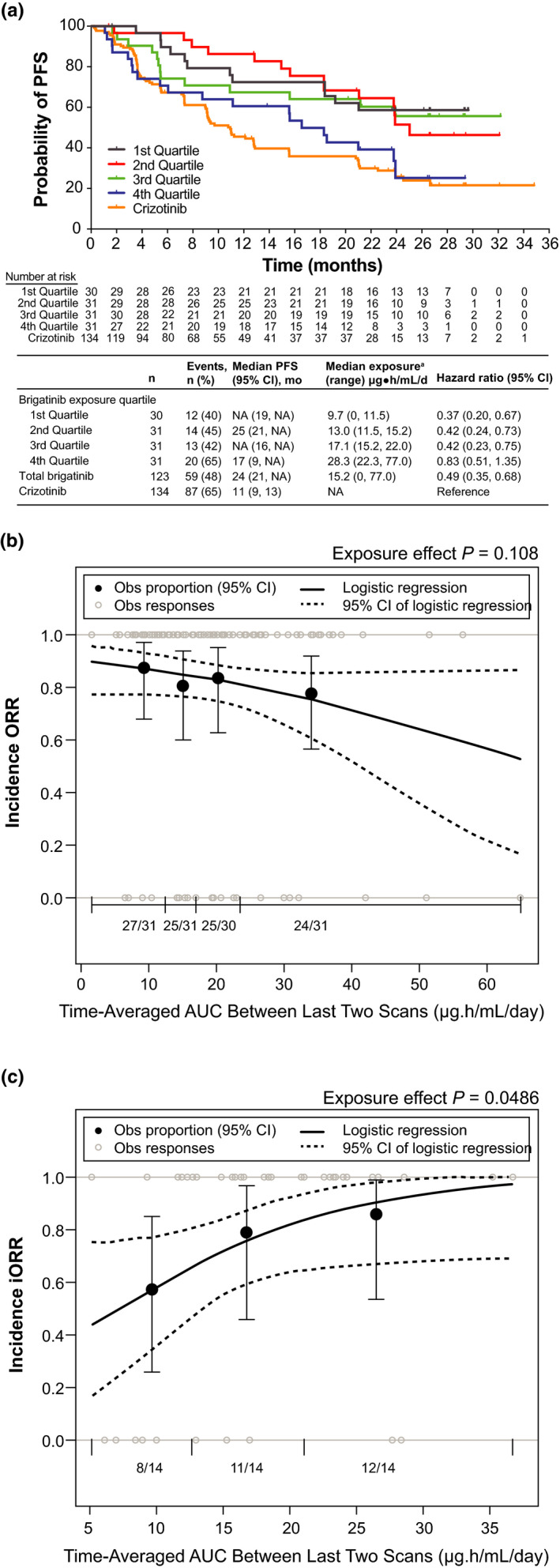
Exposure‐efficacy analyses. (a) Kaplan‐Meier probability of PFS by simulated brigatinib exposure quartiles. To evaluate the relationship between brigatinib exposure and PFS, a static exposure metric of time‐averaged AUC between the last two disease assessment scans preceding progression or censoring was used. PFS KM estimates plotted by exposure quartiles suggested that patients with higher exposure had faster onset and higher incidence of disease progression than those with lower exposure. Values for the crizotinib arm of the study are superimposed; however, no exposure values were available for crizotinib. For median PFS values, NA indicates that the probability of having no disease progression or death has not yet gone beyond 0.50 and hence the median survival time cannot be determined. ^a^Simulated exposure metric is time‐averaged AUC between the last two disease assessment scans preceding progression for PFS or censoring. Observed incidence and model‐predicted probability of (b) ORR and (c) iORR as a function of brigatinib exposure. The relationships between ORR and iORR and brigatinib exposure were analyzed using the static exposure metric of time‐averaged AUC between the last two disease assessment scans preceding best confirmed response. The probability of response was plotted against predicted exposure values, and probabilities were calculated by observed exposure quartiles or tertiles. Exposure–clinical response relationships were characterized by logistic regression models, which did not show a significant relationship between the probability of achieving ORR and time‐averaged brigatinib AUC between the last two disease assessment scans preceding the best confirmed objective response. In contrast, time‐averaged brigatinib AUC between the last two disease assessment scans preceding best confirmed intracranial response was a statistically significant predictor of iORR in patients with brain metastases at baseline. *Dotted curves* represent the 95% CI of the logistic regression model prediction. The *horizontal black line* separated by vertical black solid lines denotes the brigatinib exposure range in each quartile (ORR) and tertile (iORR). *Black dots* (vertical lines) represent the observed proportion of patients (95% CI) in each quartile (ORR) and tertile (iORR). *n*/*N* is the number of patients with events/total number of patients in each quartile (ORR) and tertile (iORR). *Grey open circles* represent observed individual data. AUC, area under the concentration‐time curve; CI, confidence interval; KM, Kaplan‐Meier; iORR, intracranial objective response rate; NA, not available; Obs, observed; ORR, objective response rate; PFS, progression‐free survival

The exposure‐efficacy analysis population included the 123 PK‐evaluable patients in ALTA‐1L (Table 1). To evaluate the relationship between brigatinib exposure and PFS, a static exposure metric of time‐averaged AUC between the last two disease assessment scans preceding progression or censoring was initially used. KM estimates of PFS plotted by exposure quartiles (Figure 2a) suggested that patients with higher exposure had faster onset and higher incidence of disease progression than those with lower exposure. The Cox PH model showed a significant relationship between the hazard of disease progression or death and time‐averaged AUC between the last two disease assessments (HR [95% CI]: 1.03 [1.01–1.05]; *p* = 0.01; i.e., there is a 3% increase in expected hazard relative to a one‐unit increase in exposure). The crizotinib arm was associated with a higher risk of disease progression compared with all brigatinib exposure quartiles (Figure 2a). Similar exposure‐PFS relationships were observed with the static metric time‐averaged AUC until progression or censoring. However, no significant relationships between PFS and exposure were identified using dynamic exposure metrics, including daily time‐averaged AUC between successive disease assessments preceding a PFS event or censoring (Cox PH ratio: 1.02 [95% CI: 1.00–1.05], *p* = 0.08) and daily time‐varying AUC preceding progression or censoring (1.01 [0.98–1.03], *p* = 0.69).

Because the analysis based on static exposure metrics identified a statistically significant effect of exposure on the hazard of disease progression or death, the impact of covariates was assessed. Only ECOG performance status was a statistically significant predictor of disease progression or death (*p* < 0.001); patients with baseline ECOG performance status 1 tended to progress faster compared with those with ECOG performance status 0. Other covariates tested were not significant predictors, including sex (*p* = 0.101), race (White vs. others, *p* = 0.86), prior chemotherapy (*p* = 0.898), baseline brain metastases (*p* = 0.781), smoking history (*p* = 0.077), age (*p* = 0.503), and sum of target lesion at baseline (*p* = 0.039). After addition of ECOG performance status to the model, the static exposure effect remained statistically significant.

Exposure‐response relationships for ORR and iORR were evaluated using two static exposure metrics. Logistic regression analyses did not show a significant relationship between the probability of achieving ORR and time‐averaged brigatinib AUC between the last two disease assessments preceding the best confirmed objective response (odds ratio: 0.97 [95% CI: 0.93–1.01], *p* = 0.108; Figure 2b) or time‐averaged AUC until the first best confirmed response (0.97 [0.94–1.01], *p* = 0.13). A significant relationship was identified between the probability of achieving iORR and time‐averaged brigatinib AUC between the last two disease assessments preceding best confirmed intracranial response (odds ratio: 1.13 [95% CI: 1.01–1.28], *p* = 0.049; Figure 2c), corresponding to estimated probabilities of response of 0.58, 0.83, and 0.99 at the 5th, 50th, and 95th exposure percentiles following 180 mg dosing. Additional covariate analysis of the iORR model did not identify any covariates as significantly affecting iORR, whereas the effect of brigatinib exposure remained significant. No significant relationship was observed between the probability of iORR and time‐averaged AUC until the first best confirmed response (1.09 [0.98–1.23], *p* = 0.15).

### Exposure‐safety analysis

The relationship between brigatinib exposure and the incidence of AEs of interest in ALTA‐1L (Table 3) was explored. Logistic regression analysis using the static exposure metric of time‐averaged brigatinib AUC to first occurrence of an event did not show a trend for a relationship with the AEs evaluated (Figures S6 and S7), with the exception of weak trends for grade ≥3 lipase increase (estimated odds ratio: 1.03 [95% CI: 0.99–1.07], *p* = 0.146) and grade ≥2 amylase increase (1.04 [0.99–1.09], *p* = 0.094).

**TABLE 3:** Incidence of grade ≥2 and grade ≥3 AEs of interest evaluated in the exposure‐safety analyses

	Brigatinib (*n* = 123)	
	Grade ≥3, *n* (%)	Grade ≥2, *n* (%)
Any AE^a^	64 (52.0)	–
Hypertension	–	38 (30.9)
CPK increase	32 (26.0)	–
Lipase increase	22 (17.9)	–
Rash	–	15 (12.2)
Amylase increase	10 (8.1)	24 (19.5)
ALT increase	6 (4.9)	18 (14.6)
AST increase	5 (4.1)	10 (8.1)
Bradycardia	–	2 (1.6)
Hyperglycemia	–	2 (1.6)
Pulmonary AEs^b^	–	2 (1.6)

Note

Data are reported as the number of patients (%).

Abbreviations: AE, adverse event; ALT, alanine aminotransferase; AST aspartate aminotransferase; CPK, creatine phosphokinase.

^a^ Any grade ≥3 AE included CPK, AST, ALT, amylase, lipase, hyperglycemia, hypertension, bradycardia, rash, and pulmonary events.

^b^ Pulmonary AEs include pneumonitis and interstitial lung disease.

**FIGURE 3:**
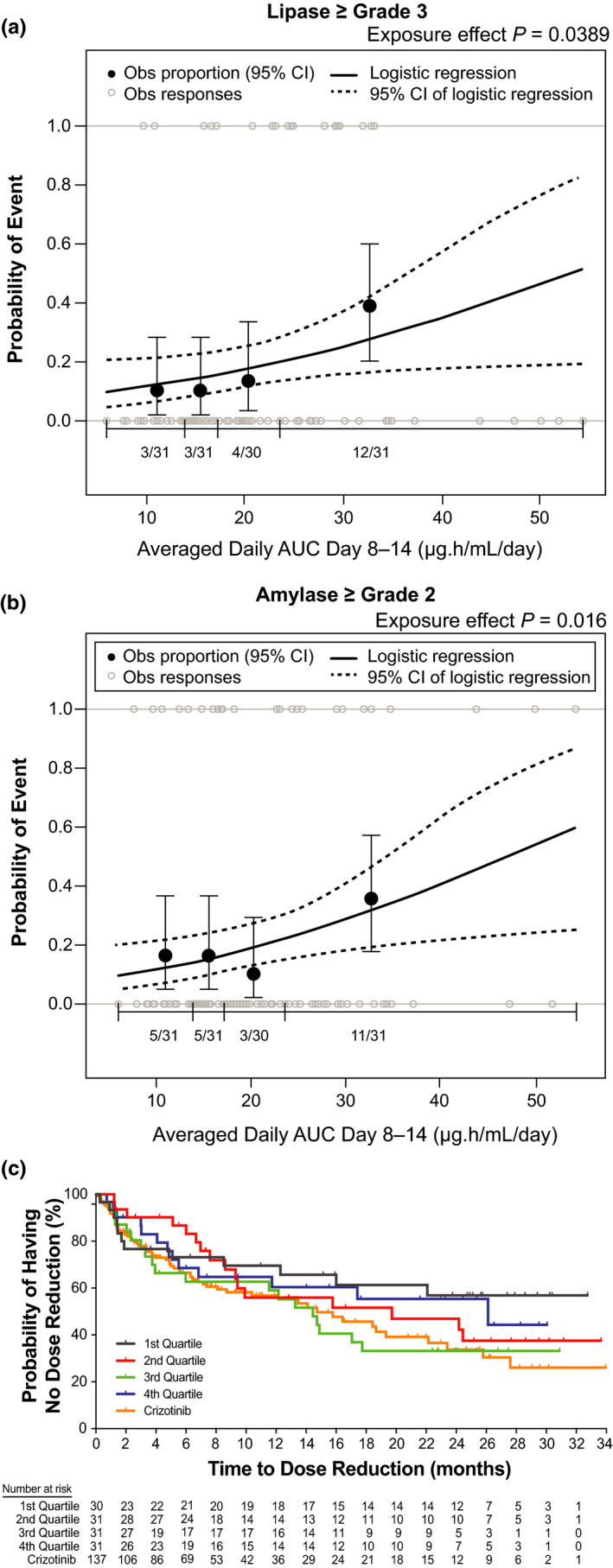
Exposure‐safety analyses. Observed incidence and predicted probability of (a) grade ≥3 lipase increase and (b) grade ≥2 amylase increase as a function of brigatinib exposure. The relationship between time‐averaged AUC across days 8 to 14 of cycle one and AE probability was examined using logistic regression models. The analysis demonstrated a statistically significant relationship between exposure and grade ≥3 lipase increase and grade ≥2 amylase increase. (c) Kaplan‐Meier estimates for time to first brigatinib dose reduction stratified by time‐averaged AUC quartiles. To explore the relationship between brigatinib exposure and dose reductions, KM plots of time to first brigatinib dose reduction were generated for brigatinib exposure (time‐averaged AUC to the first occurrence of a dose reduction) quartiles. No discernible effect of brigatinib exposure on time to first brigatinib dose reduction was noted. Values for the crizotinib arm of the study are superimposed; however, no exposure values were available for crizotinib. *Dotted curves* represent the 95% CI of the logistic regression model prediction. The *horizontal black line* separated by vertical black solid lines denotes the brigatinib exposure range in each quartile. *Black dots* (vertical lines) represent the observed proportion of patients (95% CI) in each quartile. *n*/*N* is the number of patients with events/total number of patients in each quartile. *Grey open circles* represent observed individual data. AE, adverse event; AUC, area under the concentration‐time curve; CI, confidence interval; KM, Kaplan‐Meier; Obs, observed

Use of a different static exposure metric, time‐averaged AUC across days 8 to 14 of cycle one (i.e., the week after dose increase from 90 to 180 mg q.d.) demonstrated significant relationships between exposure and grade ≥3 lipase and grade ≥2 amylase increase (Figure 3a,b). Odds ratios corresponding to an increase in brigatinib exposure of 1 µg·h/mL/day for grade ≥3 lipase and grade ≥2 amylase increases were 1.05 (95% CI: 1.00–1.10; *p* = 0.039) and 1.06 (95% CI: 1.01–1.11, *p* = 0.016), respectively.

The relationship between brigatinib time‐averaged AUC and likelihood of dose reduction was explored. KM plots of dose reduction probability and exposure quartiles did not show a discernible effect of brigatinib exposure on time to first brigatinib dose reduction (Figure 3c).

## DISCUSSION

A previously developed population PK model^11^ together with brigatinib concentration data from patients with *ALK*+ NSCLC receiving first‐line brigatinib in the ALTA‐1L trial were leveraged to obtain post hoc PK parameters. The individual‐predicted concentrations were consistent with observed concentrations from ALTA‐1L. Observed and population‐predicted ALTA‐1L PK profiles were slightly higher compared with those for second‐line treatment in ALTA, but exposures were largely overlapping. Furthermore, the absence of a trend in CWRES changes over time confirmed no apparent change in CL/F over time. Consistent with previous results,^11^ analysis of ALTA‐1L data showed no clinically meaningful effects of the covariates evaluated on brigatinib exposure, further supporting the lack of a need for brigatinib dosage adjustment for patients with mild or moderate renal or hepatic impairment,^9^ as well as based on age, sex, race, or body weight. However, for patients with severe renal and hepatic impairment, the brigatinib dose should be reduced based on results of dedicated PK studies.^9^, ^12^ ALTA‐1L was a multiregional clinical trial, and the demonstration of consistent systemic brigatinib exposures in Asian and White races (Figure S5B) provides further evidence that brigatinib PK is not sensitive to ethnic factors.^11^ Taken together with the lack of discernible exposure‐safety relationships observed in our analyses, these findings support an overall assessment of low ethnic sensitivity of brigatinib, of importance to informing scientifically guided Asia‐inclusive development and use.^17^, ^18^

Results of exposure‐PFS analyses based on static exposure metrics suggested a pharmacologically inconsistent positive relationship between increasing brigatinib exposure and risk of disease progression or death. Similar results were observed when a static exposure metric (average trough concentration) was used in exposure‐efficacy analyses of another ALK inhibitor, ceritinib.^19^ These results were attributed to patients having higher drug concentrations in earlier versus later cycles because of increased dose reductions and interruptions with longer treatment duration.^19^ In ALTA‐1L, the median time to first brigatinib dose reduction was ~ 2 years, while the median PFS appears to be 2 years or longer.^8^ Thus, patients with longer PFS have a higher likelihood of a dose reduction, in which case analyses based on time‐independent exposure metrics may lead to biased results. When time‐varying exposure metrics that better account for dose adjustments were used, brigatinib exposure was not a significant predictor of PFS. This contrasts with the previous brigatinib exposure‐response analysis of patients with refractory *ALK*+ NSCLC in the phase I/II and ALTA trials showing that increasing daily time‐varying brigatinib AUC was a significant predictor of improved PFS.^13^, ^19^ The lack of a similar exposure‐response relationship in ALTA‐1L may be attributable to the availability of data from only one dose level for the current analysis, whereas data from multiple dose levels (including 90 and 180 mg q.d. [7‐day lead‐in at 90 mg] in ALTA) were used in the earlier analysis. However, similar results were reported for ceritinib and alectinib.^19^, ^20^ Use of a time‐dependent exposure metric for ceritinib suggested a nonsignificant relationship between ceritinib exposure and risk of disease progression (HR: 1.04 [95% CI: 0.92–1.17]).^19^ For alectinib, a time‐independent measure (median observed steady‐state trough concentrations) was not a significant predictor of overall survival in patients with crizotinib‐resistant *ALK*+ NSCLC.^20^

The approaches used in our exposure‐response analyses for brigatinib in ALTA‐1L are in line with methods commonly applied in evaluation of exposure‐response relationships in oncology drug development.^21^, ^22^, ^23^, ^24^, ^25^ Nevertheless, it should be noted that a limitation of exposure‐PFS analyses is that the impact of immortal time bias remains unknown, a potential confounding factor that should be recognized in exposure‐response analyses of time‐to‐event end points. Nevertheless, the consistent lack of a discernible exposure‐related increase in PFS across the different dynamic methods/exposure metrics applied in our analyses supports a generally consistent brigatinib PFS benefit across the range of exposures achieved in this population at the recommended dosage.

In the exposure–clinical response analyses, brigatinib exposure was not a significant predictor of ORR. However, a significant relationship was observed between brigatinib exposure and iORR, with higher exposure associated with higher iORR. These results support escalation to 180 mg q.d. for patients tolerating the 7‐day lead‐in at 90 mg q.d. Brigatinib 180 mg q.d. provides substantial intracranial efficacy with high response rates that are durable.^6^ In the second ALTA‐1L interim analysis, first‐line brigatinib resulted in an iORR of 78% in patients with measurable baseline brain metastases compared with 26% for crizotinib.^8^ The 2‐year intracranial PFS rate for patients with any baseline brain metastases was 48% for brigatinib and 15% for crizotinib.

The only AEs significantly associated with brigatinib exposure were grade ≥3 lipase and grade ≥2 amylase increases. The incidences of any‐grade AEs of elevated lipase and amylase ranged from 17% to 23% and 15% to 18% in ALTA and ALTA‐1L, respectively.^4^, ^5^, ^7^, ^8^ Among other ALK inhibitors, ceritinib is associated with a higher frequency of elevated amylase and lipase compared with crizotinib and alectinib.^26^ A relationship between CPK elevations and brigatinib exposure was not identified in the exposure‐safety analyses, and elevated CPK was not associated with the frequency or severity of myalgia or musculoskeletal pain.^7^, ^8^ In the current analysis as first‐line therapy, exposure did not have a discernible relationship with time to first brigatinib dose reduction.

A potential limitation of this study is the narrow dose range used in ALTA‐1L, as all patients started brigatinib at the same dose. However, a previous exposure‐response analysis that included a broader range of doses established exposure‐response relationships for brigatinib.^13^ Although the narrow dose range in this analysis may have limited our ability to detect significant relationships, it did reveal a trend for a relationship between AE incidence and exposure. Despite the lack of a wide dose range, exposure‐response analyses can still provide important supportive evidence of a potential efficacy plateau and/or increasing toxicity with higher doses.

## CONCLUSIONS

Brigatinib PK in patients receiving first‐line brigatinib was similar to that observed for second‐line brigatinib. No significant relationship was observed between increasing brigatinib exposure and improved PFS or ORR. However, higher brigatinib exposure was associated with higher intracranial ORR. With the exceptions of grade ≥3 lipase and grade ≥2 amylase elevations, there were no significant relationships between brigatinib exposure and AE incidence or time to first dose reduction.

## CONFLICT OF INTEREST

Neeraj Gupta: Employment (Takeda). Karen L Reckamp: Consultant/honoraria (to self) (Calithera, Euclises, Guardant, Precision Health, Amgen, AstraZeneca, Blueprint, Boehringer Ingelheim, Daiichi Sankyo, EMD Serono, Genentech, Janssen, Lilly, Merck KGaA, Seattle Genetics, Takeda, Tesaro; grant/research support (AbbVie, ACEA, Adaptimmune, Guardant, Molecular Partners, Seattle Genetics, Boehringer Ingelheim, Bristol‐Myers Squibb, Genentech, GlaxoSmithKline, Janssen, Loxo Oncology, Spectrum, Takeda, Xcovery, Zeno, Calithera, Daiichi Sankyo, Elevation Oncology). D. Ross Camidge: Honoraria (AstraZeneca, Takeda, Arrys/Kyn, Genoptix, G1 Therapeutics (DSMB), Mersana Therapeutics, Roche/Genentech, Ignyta, Daiichi Sankyo (ILD adjudication committee), Hansoh SRC, Bio‐Thera DSMB, Lycera, Revolution Med, Orion, Clovis, Celgene, Novartis); research funding: Ariad/Takeda). Huub Jan Kleijn: Consultant (Certara). Aziz Ouerdani: Consultant (Certara). Francesco Bellanti: Consultant (Certara). John Maringwa: Consultant (Certara). Michael J. Hanley: Employment (Takeda). Shining Wang: Employment (Takeda). Pingkuan Zhang: Employment (Takeda). Karthik Venkatakrishnan: Former employee (Takeda); current employee (EMD Serono Research & Development Institute, Inc.).

## AUTHOR CONTRIBUTIONS

N.G. and M.J.H. wrote the manuscript. N.G., H.J.K., and K.V. designed the research. H.J.K., A.O., F.B., and J.M. performed the research. N.G., K.L.R., D.R.C., H.J.K., A.O., F.B., J.M., M.J.H., S.W., P.Z., and K.V. analyzed the data.

## Supporting information

Figure S1

Figure S2

Figure S3

Figure S4

Figure S5

Figure S6

Figure S7

Supplementary Material
