# Supplementary material for: Population pharmacokinetic and exposure‐response analyses from ALTA‐1L: Model‐based analyses supporting the brigatinib dose in ALK‐positive NSCLC
Source: Clin Transl Sci. 2022 Feb 8;15(5):1143–54. doi: 10.1111/cts.13231 (PMC9099121; doi:10.1111/cts.13231)
Supplement: Supplementary file 1 — Figure S1 [file CTS-15-1143-s006.pdf]

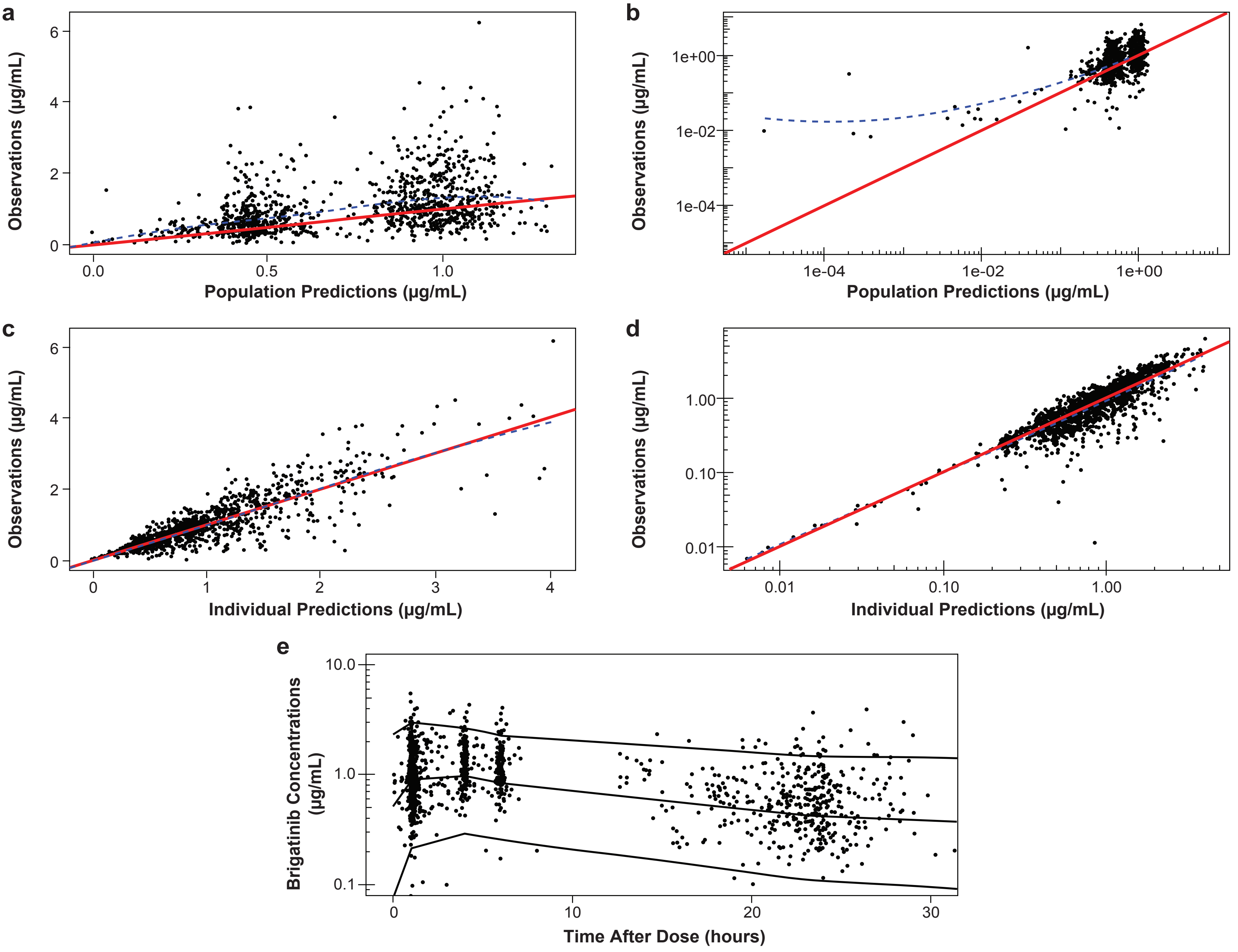

Figure S1. ALTA-1L observed concentrations versus population-predicted plasma brigatinib concentrations in **a** normal and **b** log scale and observed concentrations versus individual predicted plasma brigatinib concentrations in **c** normal and **d** log scale. **e** Prediction-corrected visual predictive check comparing model-simulated values to the observed data in ALTA-1L. Population and individual predicted brigatinib concentrations were plotted against observed concentrations in ALTA-1L to evaluate general model fit to the observed data. The distribution of patient-level random effects estimated for patients was compared between patients from ALTA-1L and ALTA. Prediction-corrected visual predictive checks were generated to evaluate the ability of the population PK model to describe the mean and variability of individual dose-normalized brigatinib concentration-time profiles. These prediction-corrected visual predictive checks suggested general agreement between predicted and observed brigatinib concentrations. For **a** to **d**, *black dots* are observed brigatinib concentrations. Points are individual data, *dashed lines* represent the locally weighted scatterplot smoothing (loess) fitting, and *solid red diagonal lines* are the line of unity. For **e**, *black dots* are observed data. *Black lines* from top to bottom are 97.5th percentile, median, and 2.5th percentile of the brigatinib PK profiles simulated from 1000 replicates.

PK pharmacokinetic
