# Supplementary material for: Population pharmacokinetic and exposure‐response analyses from ALTA‐1L: Model‐based analyses supporting the brigatinib dose in ALK‐positive NSCLC
Source: Clin Transl Sci. 2022 Feb 8;15(5):1143–54. doi: 10.1111/cts.13231 (PMC9099121; doi:10.1111/cts.13231)
Supplement: Supplementary file 2 — Figure S2 [file CTS-15-1143-s001.pdf]

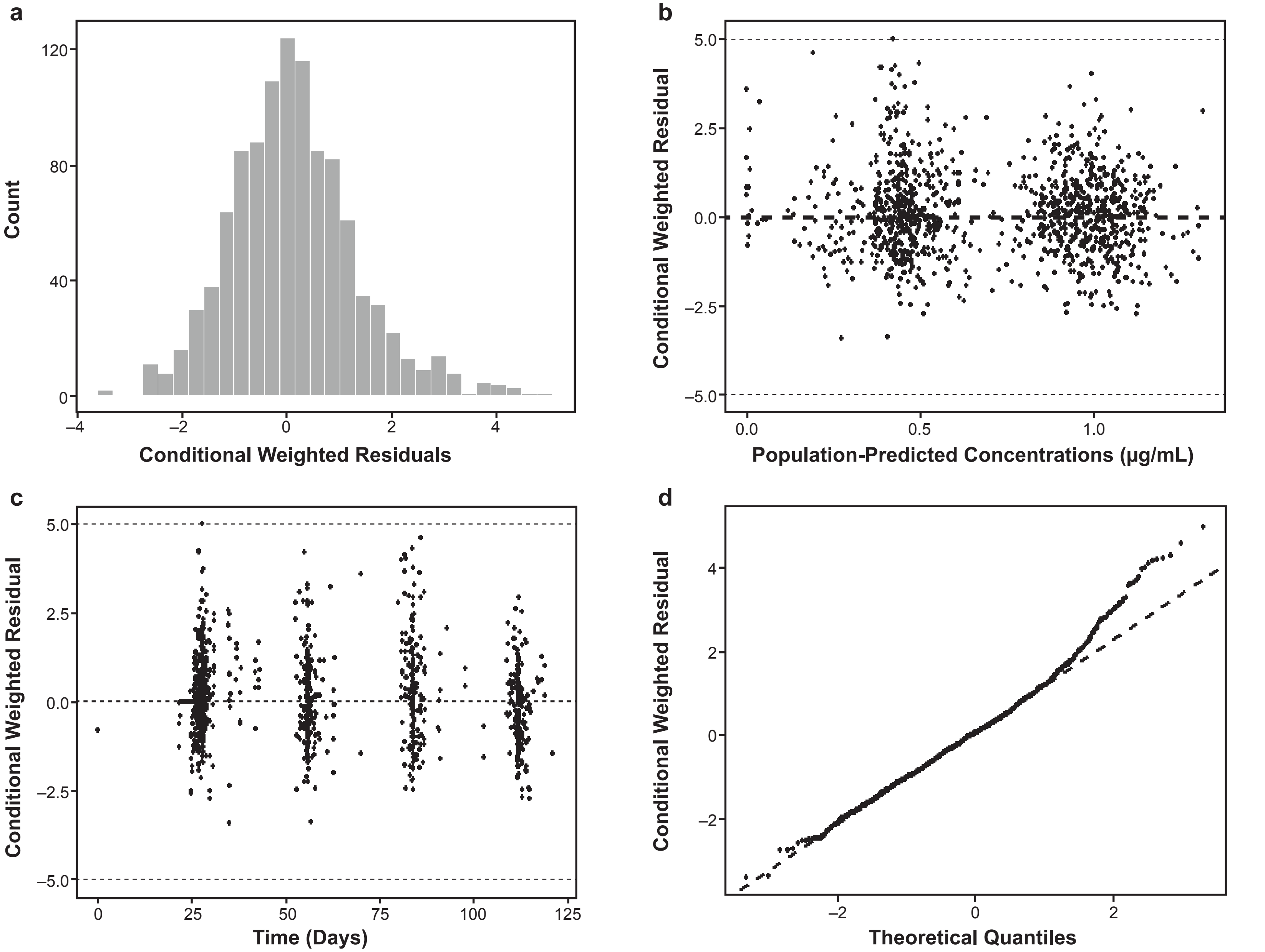

Figure S2. Residual-based diagnostics of the population PK model applied to first-line brigatinib in ALK+ NSCLC patients in ALTA-1L. **a** Distribution of conditional weighted residuals (CWRES). **b** CWRES versus population predictions. **c** CWRES versus time. **d** Q-Q plot comparing the distribution of CWRES to a normal distribution. The CWRES were normally distributed and showed no trend when related to population-predicted concentrations or time, further supporting the ability of the model to describe the ALTA-1L data.

ALK+ NSCLC anaplastic lymphoma kinase-positive non–small cell lung cancer
