# Supplementary material for: Population pharmacokinetic and exposure‐response analyses from ALTA‐1L: Model‐based analyses supporting the brigatinib dose in ALK‐positive NSCLC
Source: Clin Transl Sci. 2022 Feb 8;15(5):1143–54. doi: 10.1111/cts.13231 (PMC9099121; doi:10.1111/cts.13231)
Supplement: Supplementary file 3 — Figure S3 [file CTS-15-1143-s008.pdf]

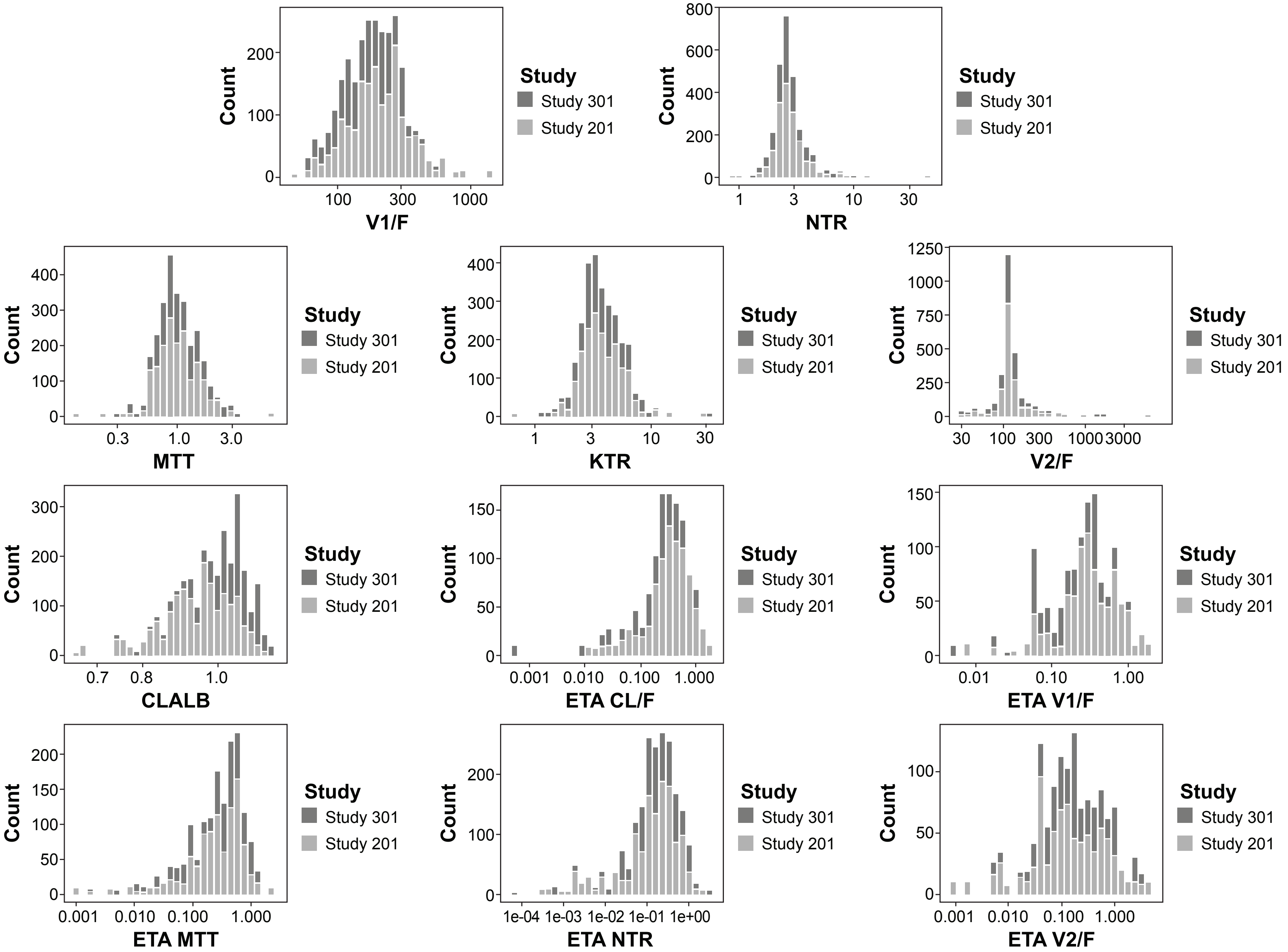

Figure S3. Histograms comparing individual PK parameter estimates between ALTA-1L and ALTA. To further justify the ability of the model to accurately predict exposure in patients in ALTA-1L, distribution of post hoc PK estimates were compared for patients in ALTA and ALTA-1L. The distributions of individual PK parameters were similar between the trials, confirming that the Bayesian re-estimation approach was an appropriate method to obtain individual PK parameters and post hoc exposure estimates for ALTA-1L.

CLALB effect of albumin level on apparent oral clearance, *ETA CL/F* interindividual variability on apparent oral clearance, *ETA MTT* interindividual variability on mean transit time, *ETA NTR* interindividual variability on number of transit compartments, *ETA V1/F* interindividual variability on apparent central volume of distribution, *ETA V2/F* interindividual variability on apparent volume of distribution in the first peripheral compartment, *KTR* transfer rate constant between the transit compartments, *MTT* mean transit time, *NTR* number of transit compartments, *PK* pharmacokinetic, *V1/F* apparent central volume of distribution, *V2/F* apparent volume of the first peripheral compartment
