# Supplementary material for: Population pharmacokinetic and exposure‐response analyses from ALTA‐1L: Model‐based analyses supporting the brigatinib dose in ALK‐positive NSCLC
Source: Clin Transl Sci. 2022 Feb 8;15(5):1143–54. doi: 10.1111/cts.13231 (PMC9099121; doi:10.1111/cts.13231)
Supplement: Supplementary file 4 — Figure S4 [file CTS-15-1143-s003.pdf]

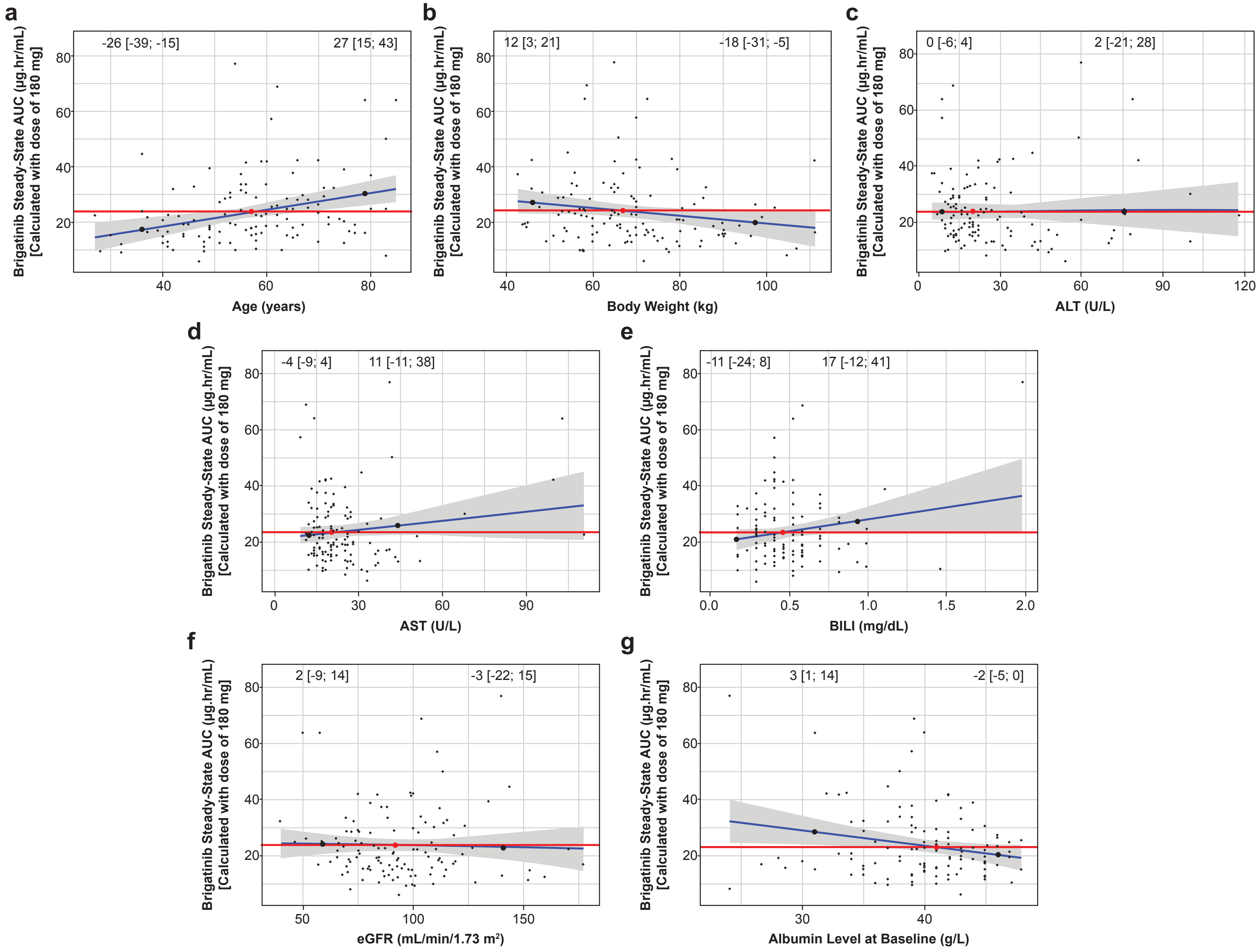

Figure S4. Correlations between individual predicted steady-state brigatinib exposure (AUC) after 180 mg once daily and continuous covariates of **a** age, **b** body weight, **c** ALT levels, **d** AST levels, **e** total bilirubin, **f** eGFR, and **g** albumin were explored using linear regression models, with each covariate as a predictor. None of the covariates examined had clinically meaningful effects on brigatinib exposure in ALTA-1L. In panels **a–g**, *red* and *black dots* represent the median and the 5th and 95th percentiles of the individual covariate values, respectively, *numbers (brackets)* are the percentage change in the AUC at the 5th and 95th percentiles relative to the values at the median, based on the linear regression (*blue line*) and corresponding 95% CI (*shaded region*), and the red line is the horizontal line passing through the AUC value corresponding to the median covariate value in the population. *ALT* alanine aminotransferase, *AST* aspartate aminotransferase, *AUC* area under the concentration-time curve, *BILI* bilirubin, *CI* confidence interval, *eGFR* estimated glomerular filtration rate
