# Supplementary material for: Population pharmacokinetic and exposure‐response analyses from ALTA‐1L: Model‐based analyses supporting the brigatinib dose in ALK‐positive NSCLC
Source: Clin Transl Sci. 2022 Feb 8;15(5):1143–54. doi: 10.1111/cts.13231 (PMC9099121; doi:10.1111/cts.13231)
Supplement: Supplementary file 5 — Figure S5 [file CTS-15-1143-s004.pdf]

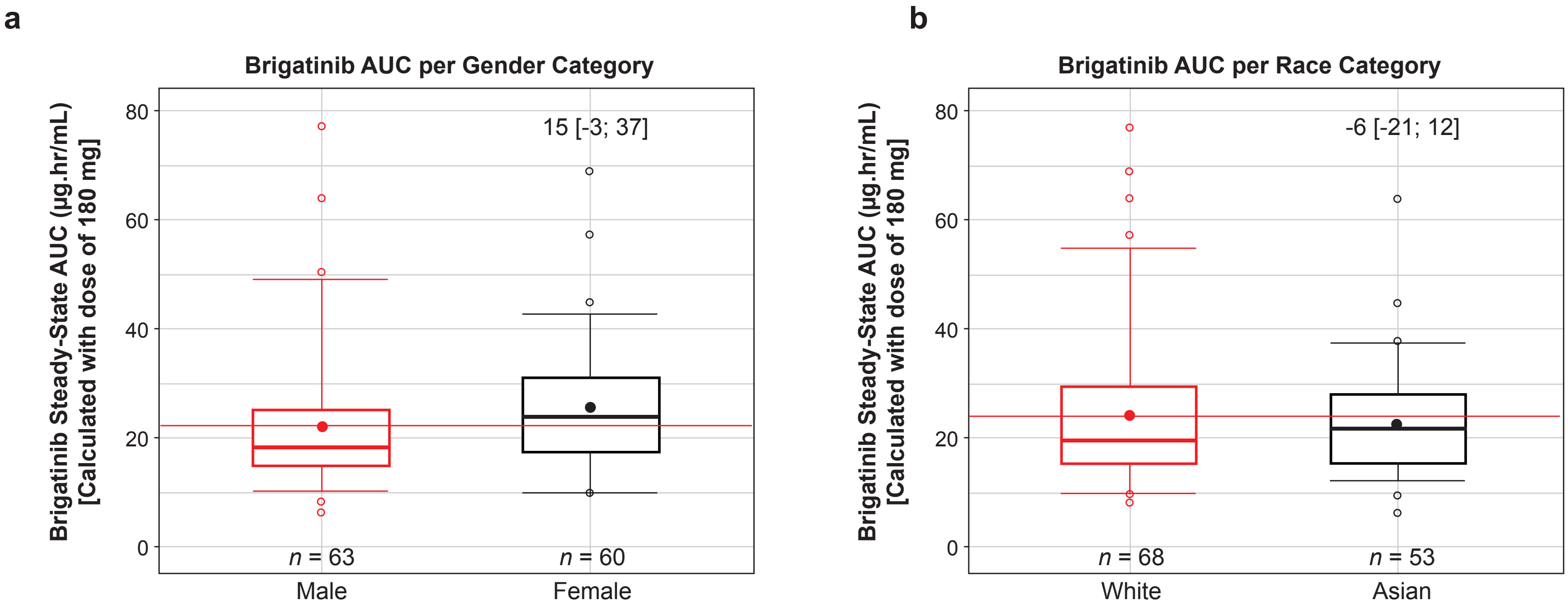

Figure S5. Correlations between individual predicted steady-state brigatinib exposure (AUC) after 180 mg once daily and categorical covariates of **a** sex and **b** race were explored using linear regression models,with each covariate as a predictor. Neither covariate examined had clinically meaningful effects on brigatinib exposure in ALTA-1L. In panels **a–b**, *red* and *black dots* represent the arithmetic mean exposure in the reference category and the remaining category, respectively, and *numbers (brackets)* are the percentage change in median AUC (with 95% CI) relative to the most common reference category of the individual covariate values, respectively. Numbers at the bottom of the boxplot hinge represent the sample size in each category. For the race covariate, the category “unknown” was removed.

AUC area under the concentration-time curve, *CI* confidence interval
