# Supplementary material for: Population pharmacokinetic and exposure‐response analyses from ALTA‐1L: Model‐based analyses supporting the brigatinib dose in ALK‐positive NSCLC
Source: Clin Transl Sci. 2022 Feb 8;15(5):1143–54. doi: 10.1111/cts.13231 (PMC9099121; doi:10.1111/cts.13231)
Supplement: Supplementary file 7 — Figure S7 [file CTS-15-1143-s005.pdf]

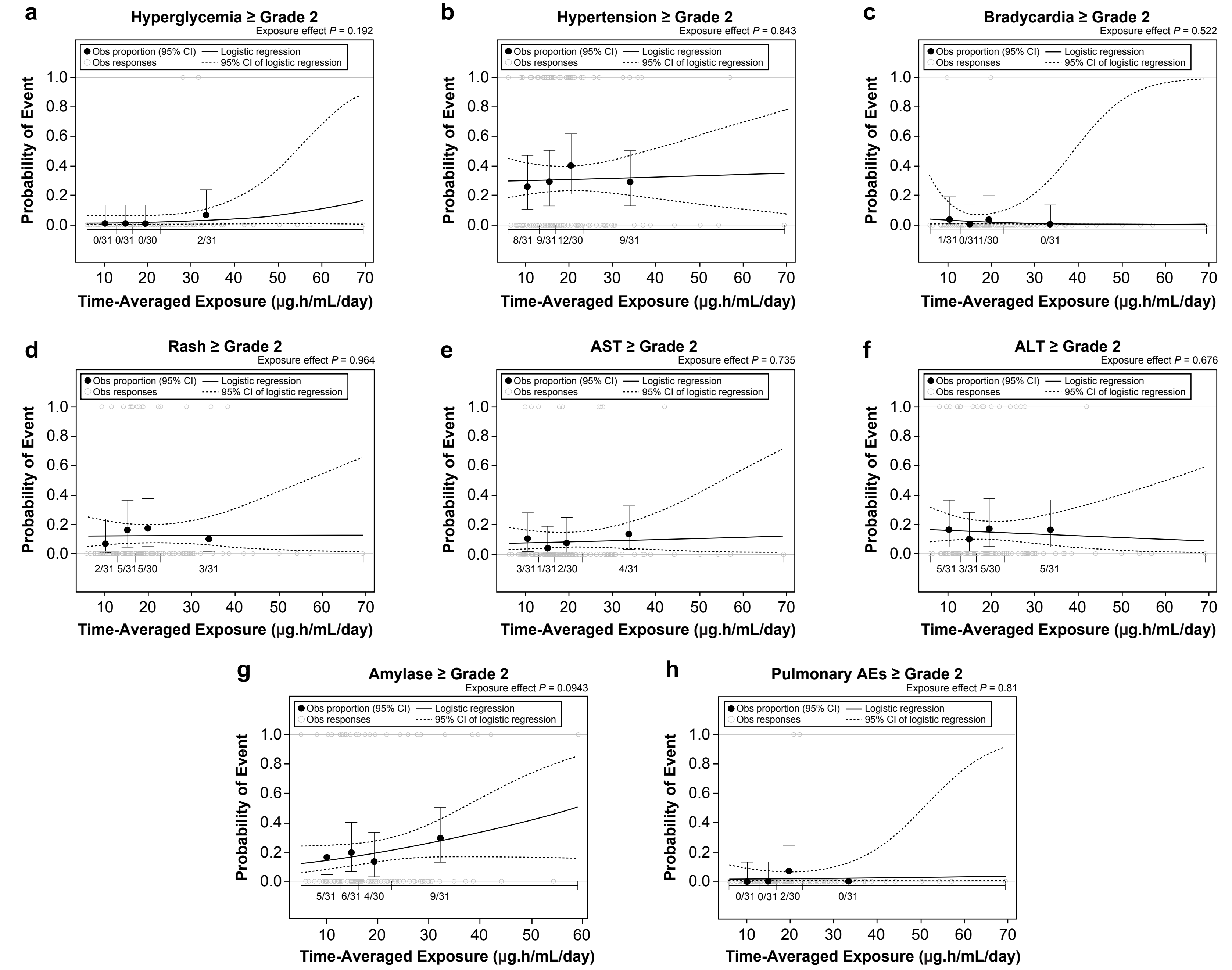

Figure S7. Observed incidence and predicted probability of grade ≥ 2 AEs of interest as a function of time-averaged brigatinib AUC to the first occurrence of an event or the end of treatment in case of no event using a logistic regression model. AEs were included in the analysis if they occurred from the first day of brigatinib dosing until 30 days after the last dose. The relationship between time-averaged exposure and AE probability was examined using the same logistic regression models described for the exposure–clinical response analyses. No clear trend was shown for a relationship with any of the AEs evaluated, with the exception of a weak trend for grade ≥ 2 amylase increase.

AE adverse event, *ALT* alanine aminotransferase, *AST* aspartate aminotransferase, *AUC* area under the concentration-time curve, *CI* confidence interval, *OBS* observed
