# Supplementary material for: Population pharmacokinetic and exposure‐response analyses from ALTA‐1L: Model‐based analyses supporting the brigatinib dose in ALK‐positive NSCLC
Source: Clin Transl Sci. 2022 Feb 8;15(5):1143–54. doi: 10.1111/cts.13231 (PMC9099121; doi:10.1111/cts.13231)
Supplement: Supplementary file 8 — Supplementary Material [file CTS-15-1143-s007.docx]

# ELECTRONIC SUpplementary Material

**Methods S1.**

**Derivation of exposure metrics**

The following time-averaged AUCs were considered in exposure-efficacy analyses:

1. Dynamic daily time-averaged AUC between successive disease assessment scans preceding the event or censoring for PFS, obtained as the average of daily AUCs within each scan interval.

Daily AUC was based on the cumulative AUC to day *i* (${CAUC}_{i}$) defined as:

${CAUC}_{i}= \frac{Cumulative dose to day i}{Oral brigatinib clearance}$

The daily AUC at day i ($AUCD_{i}$) was defined as:

$AUCD_{i}= {CAUC}_{i+1}- {CAUC}_{i}$

Where daily AUC was derived by Bayesian estimation of rich concentration-time profiles over the treatment duration (or to the event) and where AUC is calculated for each dosing interval.

1. Static metrics of time-averaged AUC until PFS, ORR, or iORR (or censoring), defined as:

$$Time averaged AUC until PFS= \frac{Cumulative dose to progression}{Oral brigatinib clearance}/ (days to progression)$$

$$Time averaged AUC until best confirmed response (ORR or iORR)= \frac{Cumulative dose to best confirmed systemic \left( \mathrm{ORR} \right) or intracranial \left( \mathrm{iORR} \right)\mathrm{response}}{Oral brigatinib clearance}/ (days to best confirmed systemic or intracranial response)$$

In cases of no PFS or response events, the time-averaged AUC until censoring (end of treatment) was defined as:

$$\frac{Cumulative dose}{Oral brigatinib clearance}/ (Total days of treatment)$$

1. Static metrics of time-averaged exposure between the last two disease assessment scans preceding PFS, ORR, or iORR, or censoring (refer to 2)
